# MCP‐1 downregulates MMP‐9 export via vesicular redistribution to lysosomes in rat portal fibroblasts

**DOI:** 10.14814/phy2.12153

**Published:** 2014-11-20

**Authors:** DaShawn A. Hickman, Gaurav Syal, Michel Fausther, Elise G. Lavoie, Jessica R. Goree, Brian Storrie, Jonathan A. Dranoff

**Affiliations:** 1 Division of Gastroenterology and Hepatology, Department of Internal Medicine, University of Arkansas for Medical Sciences, Little Rock, Arkansas; 2 Department of Physiology and Biophysics, University of Arkansas for Medical Sciences, Little Rock, Arkansas

**Keywords:** Lysosome, MCP‐1, MMP‐9, portal fibroblast

## Abstract

Portal fibroblasts (PF) are one of the two primary cell types contributing to the myofibroblast population of the liver and are thus essential to the pathogenesis of liver fibrosis. Monocyte chemoattractant protein‐1 (MCP‐1) is a known profibrogenic chemokine that may be of particular importance in biliary fibrosis. We examined the effect of MCP‐1 on release of matrix metalloproteinase‐9 (MMP‐9) by rat PF. We found that MCP‐1 blocks PF release of MMP‐9 in a posttranslational fashion. We employed an optical and electron microscopic approach to determine the mechanism of this downregulation. Our data demonstrated that, in the presence of MCP‐1, MMP‐9‐containing vesicles were shunted to a lysosome‐like compartment. This is the first report of a secretory protein to be so regulated in fibrogenic cells.

## Abstract (precis)

Portal fibroblasts are resident liver cells that contribute to liver fibrosis. MCP‐1 induces profibrogenic changes in portal fibroblasts. Here, we found that MCP‐1 also downregulates function of the matrix metalloproteinase MMP9 via shunting of vesicles to a lysosomal compartment.

## Introduction

Cirrhosis is the most important cause of liver failure and a major cause of mortality worldwide (Ratib et al. 2014). It has long been clear that there are two distinct varieties of cirrhosis: biliary and nonbiliary. Nonbiliary cirrhosis is caused by such conditions as chronic viral hepatitis, alcoholic liver disease, and nonalcoholic fatty liver disease and accounts for the majority of adult cirrhosis. Biliary cirrhosis, in contrast, is caused in general by cholangiopathies – conditions targeting bile ducts, such as biliary atresia, primary biliary cirrhosis, and primary sclerosing cholangitis. While biliary cirrhosis accounts for only one in five cases of liver failure in adults, it is the predominant cause of liver failure in children (Alpini et al. 2002).

Biliary cirrhosis has long been known to be distinct in several ways: it progresses rapidly and is associated with more severe presinusoidal portal hypertension (Heathcote 1997). More recent evidence shows that the pathogenesis of biliary cirrhosis is distinct from that of nonbiliary cirrhosis. Specifically, biliary cirrhosis is mediated in large part by portal fibroblasts (PF) as effector cells, as opposed to hepatic stellate cells (HSC) alone (Beaussier et al. 2007). In the setting of biliary injury, portal fibroblasts differentiate into myofibroblasts that release matrix and profibrogenic components (Tang et al. 1994). Furthermore, the process of profibrogenic myofibroblastic differentiation of PF is directed in large part by release of bioactive mediators by cholangiocytes (Rachfal and Brigstock 2003; Kruglov et al. 2006). One of the best characterized of these mediators is CCL2/MCP‐1, which has been shown to mediate cholangiocyte‐to‐PF signaling directly (Kruglov et al. 2006).

Fibrosis is mediated by a change in equilibrium between anti and profibrogenic components secreted by cells (Iredale 1997). Antifibrotic components include matrix metalloproteinases (MMPs) (Iimuro and Brenner 2008), whereas profibrogenic components include tissue inhibitors of metalloproteinases and matrix components themselves (Ramachandran and Iredale 2009). CCL2 has been shown to modulate MMP‐9 (MacLauchlan et al. 2009), which is a factor that is relevant to liver fibrosis resolution (Ramachandran et al. 2012). With this in mind, the goals of this study were to test the effects of CCL2 on MMP‐9 release in PF. We found that CCL2 blocks MMP‐9 release by a surprising mechanism – by shunting CCL2‐containing vesicles to a lysosome‐like compartment, thus blocking exocytosis of preformed vesicles. We believe that this mechanism may be relevant not only to the phenomena observed, but also to fundamental mechanisms regulating liver fibrosis and regulated exocytosis.

## Materials and Methods

### PF isolation and culture

Rat PF were isolated as described previously (Kruglov et al. 2002). Male adult Sprague‐Dawley rats (180–300 g) were used for all experiments. Briefly, nonparenchymal cell (NPC) fractions were obtained by in situ pronase/collagenase perfusion of livers. Rat NPC were subsequently used for PF isolation by serial mesh filtration of the hilar remnant. Resulting cell suspensions were plated onto tissue culture plastic dishes or glass coverslips within tissue culture dishes in DMEM/F‐12 containing 10% FCS, and antibiotics. For the current studies, PF were used at 2–7 days after plating, at which time PF are known to be myofibroblastic (Jhandier et al. 2005; Yu et al. 2008). All cells were maintained at 37°C, under 95% air/5% CO_2_.

### Detection of functional MMP‐9 by gelatin zymography

MMP‐9 enzymatic activity was determined by SDS‐PAGE gelatin zymography (Sakalihasan et al. 1996). Supernatants from both control and MCP‐1–treated PF cultures were collected and analyzed for released (extracellular) MMP‐9 content. Conditioned media samples were denatured in Laemmli sample buffer containing 62.5 mmol/L Tris (pH 6.8), 10% (v/v) Glycerol, 2% SDS, 0.0025% Bromophenol blue (in nonreducing conditions and without heating), and electrophoresed in 7.5% SDS‐polyacrylamide gels containing 0.1% (w/v) porcine skin gelatin. Recombinant MMP‐9 (1 ng) was used as a positive control. Gels were incubated in a 2.5% Triton X‐100 solution at room temperature for 1 h and subsequently in a buffer containing 50 mmol/L Tris, 200 mmol/L NaCl, 5 mmol/L CaCl_2_, 0.02% (v/v) Brij 35 solution (pH 7.5), at 37°C for up to 48 h depending on the enzyme concentration in the analyzed sample. Afterward, gels were stained with Coomassie brilliant blue for 1 h and destained with a 40% (v/v) methanol and 10% (v/v) acetic acid solution for 30 min. MMP‐9 gelatinolytic activity (detected on gel as a white band against a blue background) was determined by densitometric scanning and quantitative analysis using the Odyssey Infrared Imaging System (Li‐Cor Biotechnology, Lincoln, NE).

### Immunoblot

Intracellular MMP‐9 protein contents in PF were determined by immunoblot. Day 7 PF plated in T‐25 tissue culture flasks were treated with 0.1–100 ng/mL MCP‐1 (Thermo Scientific, Rockford, IL) overnight or were treated with buffer alone. Following treatment, cells were scraped in 4°C PBS, and obtained protein lysates were resuspended in Laemmli sample buffer, sonicated, heat‐denatured, and electrophoresed in 7–15% tris HCl Ready Made gels (Biorad, Hercules, CA). Resolved proteins were transferred to a PVDF membrane (Immobilon/Millipore, Bedford, MA). Membranes were blocked using Odyssey Blocking Buffer (Li‐Cor Biotechnology) and incubated with a rabbit monoclonal antibody against MMP‐9 (1:1000) (Abcam, Cambridge, MA) followed by a 680 IRDYE goat anti‐rabbit secondary antibody (1:8000) (Li‐Cor Biotechnology) and imaged using the Odyssey Imaging System fluorescence scanner (Li‐Cor Biotechnology). After restoring the membranes, they were incubated with a commercial mouse monoclonal antibody against beta actin (1:5000) followed by 800 IRDYE goat anti‐mouse (1:8000) (Li‐Cor Biotechnology). In a separate set of experiments, Day 7 PF were treated with BAPTA/AM (10 *μ*mol/L), MCP‐1 (10 ng/mL), BAPTA/AM + MCP‐1, or ionomycin (10 *μ*mol/L) (Calbiochem, San Diego, CA) for 10 min before harvesting cells and following the protocol above.

### Quantitative RT‐PCR

Alterations in MMP‐9 mRNA expression following MCP‐1 treatment were determined by quantitative RT‐PCR, using GAPDH as a control gene. After treatment with 10 ng/mL of MCP‐1 overnight, PF were scraped in 4°C PBS and homogenized using QIAshredder columns (Qiagen, Valencia, CA), and total RNA was extracted using a RNeasy Micro kit (Qiagen). Reverse transcription of isolated total RNA samples was performed with QuantiTect Reverse Transcription Kit (Qiagen). PCR was performed using MMP‐9‐specific fluorescent primers using an ABI PRISM 7500 Sequence Detection System thermal cycler (Applied Biosystems, Foster City, CA).

### Confocal immunofluorescence

For confocal immunofluorescence experiments, PF were isolated as described above and plated on glass coverslips. Cells were treated as described in the prior sections, rinsed in PBS, and fixed with acetone at 4°C for 20 min. Cells were blocked with a blocking solution containing PBS, 0.1% Triton‐X, 1% BSA, and 5% goat serum for 20 min at room temperature, incubated with a goat polyclonal antibody against MMP‐9 (1/200; Santa Cruz, Dallas, TX) and a rabbit polyclonal antibody against LAMP 1 (1:400) (Abcam) for 45 min at room temperature, rinsed, then incubated with TOPRO‐3 (1:400) (Life Technologies, Grand Island, NY), Alexa Fluor 488 anti goat (1:1000), and Alexa Fluor 555 anti rabbit (1:500). For some samples, staining with rhodamine phalloidin (1:40) was performed. The slides were viewed using a Zeiss 710 Duo Confocal Microscope (Carl Zeiss Microscopy, Thornwood, NY) equipped with an Ar and He‐Ne laser.

### MMP‐9‐CFP vector

MMP‐9 cDNA was amplified from whole liver cDNA by PCR and cloned into the TOPO‐TA vector (Invitrogen, Grand Island, NY). The MMP‐9 cDNA was subcloned into a commercial C‐terminal mammalian CFP expression vector (Clontech Laboratories, Mountain View, CA). The resultant MMP‐9‐CFP construct was verified by automated sequencing. Expression of MMP‐9‐CFP or CFP alone (control) was accomplished by transient transfection of Day 5 PF using TransMessenger Transfection Reagent (Qiagen), and cells were visualized at 48 h, so that cells were at day 7 during observation.

### Live cell confocal microscopy

Live day 7 PF cells transfected with MMP‐9‐CFP or CFP were incubated with 50 nmol/L Lysotracker Red (Invitrogen) for 30 min at 37°C. Cells were then imaged with live cell confocal microscopy with a Zeiss 710 Duo Confocal Microscope (Carl Zeiss Microscopy).

### Immunoelectron microscopy

Immuno‐EM was performed as described previously (Storrie et al. 1998). Day 7 PF were plated onto T‐25 cell flasks, washed with 4°C PBS, and scraped from the bottom of the flask. Cells were pelleted by centrifuging at 2500 g for 10 min at 4°C, resuspended in 4% gluteraldehyde, and incubated for 1 h. Cells were then pelleted by centrifugation at 2500 g for 10 min at 4°C, rinsed with PBS, resuspended in 1% OsO_4_, incubated for 1 h, and rinsed with ddH_2_O. Cells were then serially dehydrated by incubating for 10 min in 70% ethanol, for 10 min in 80 ethanol, for 10 min in 90% ethanol and finally for 10 min in 100% ethanol two times. Cells were then incubated with propylene oxide for 5 min three times. Cells were then embedded with 2:1 propylene oxide:epoxy resin, 1:1 propylene oxide:epoxy resin, 1:2 propylene oxide:epoxy resin then pure epoxy resin for 30 min. A vacuum was pulled for 10 min at room temperature then the resin was cured at 60°C overnight. Samples were sectioned with microtome and imaged with an electron microscope. Immunolabeling was performed using anti‐MMP (1:100) and gold‐conjugated anti‐rabbit secondary antibodies (1:100). After immunolabeling, sections were positively stained and embedded with 2% methyl cellulose containing 0.3% uranyl acetate air‐dried, and viewed using an FEI Tecnai F20 200 keV microscope (Hillsboro, OR).

## Results

### MCP‐1 regulates PF MMP‐9 release in a posttranslational fashion

**Figure 1.**
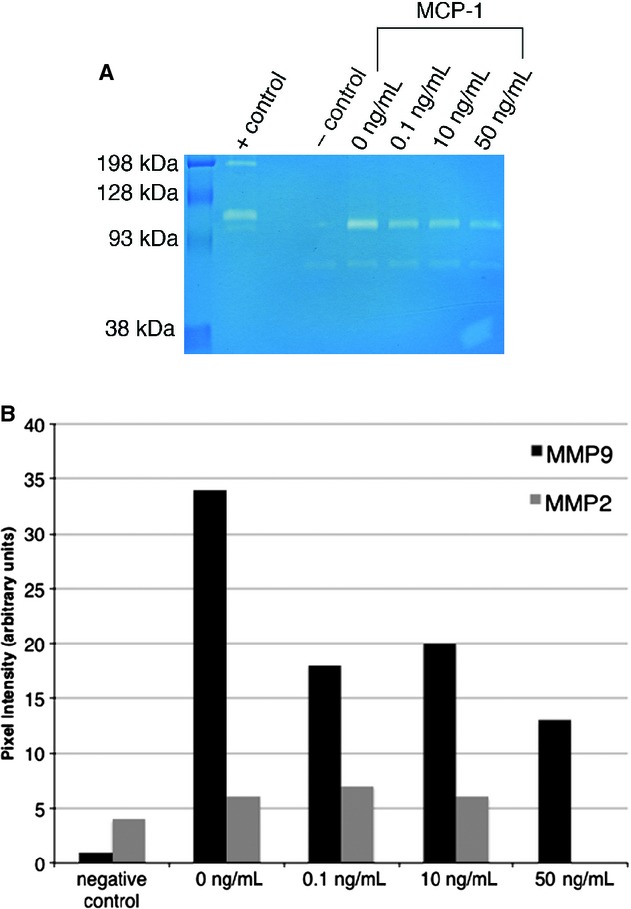
Effect of MCP‐1 on PF supernatant MMP‐9 activity as determined by gelatin zymography. (A) Representative zymogram. Primary rat PF were isolated and either left untreated or were treated overnight with MCP‐1 (0.1–50 ng/mL). Cells were washed, and the cell supernatant was collected after 30 min. As can be seen, the relative activity of MMP‐9 (seen at 92 KDa) decreases after MCP‐1 treatment. In contrast, MMP2 activity (seen at 72 KDa) is unaltered. (B) Quantitation of data. Band intensity was quantitated electronically, and changes in band intensity are shown. MCP‐1 decreased MMP‐9 band intensity to 38–53% relative to untreated cells.

**Figure 2.**
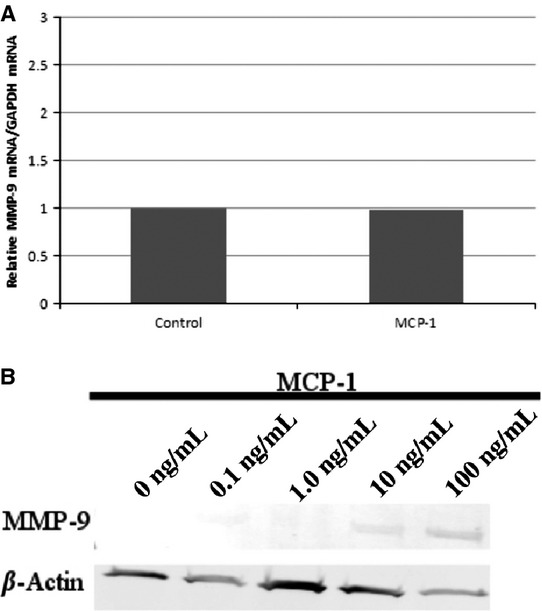
Effect of MCP‐1 on MMP‐9 mRNA and protein in PF. (A) Real‐time qRT‐PCR. PF were untreated or treated overnight with MCP‐1 (10 ng/mL), and changes in MMP‐9 mRNA were determined by qRT‐PCR. No change in MMP‐9 mRNA was noted. (B) Immunoblot. PF were treated overnight with MCP‐1 (0–100 ng/mL), cells were washed, and the supernatant was removed. Changes in MMP‐9 protein from PF lysates were detected by immunoblot. MMP‐9 protein bands at 92 KDa were detected only after treatment with MCP‐1 (10 or 100 ng/mL).

We investigated the effect of MCP‐1 on MMP‐9 activity via gelatin zymography. As seen in Figure 1, MCP‐1 decreased MMP‐9 activity in PF in a concentration‐sensitive fashion. Note that no change in MMP‐2 activity was observed, consistent with the concept that, in general, MMP‐9 is regulated (Hatfield et al. 2010), whereas MMP2 is constitutive (Gioia et al. 2009). We then examined whether the observed downregulation of MMP‐9 activity was due to downregulation of transcription. As seen in Figure 2A, MCP‐1 induced no change in MMP‐9 mRNA. Finally, we investigated whether MCP‐1 regulated the intracellular protein content of MMP‐9. We found that MMP‐9 protein could be detected in PF only after treatment with MCP‐1 (10 or 100 ng/mL). Taken together, these findings suggested that MCP‐1 altered PF MMP‐9 activity in a fashion independent of transcriptional changes and possibly due to alteration of protein release into the extracellular environment.

### MCP‐1 induces formation of MMP‐9 clusters in PF

**Figure 3.**
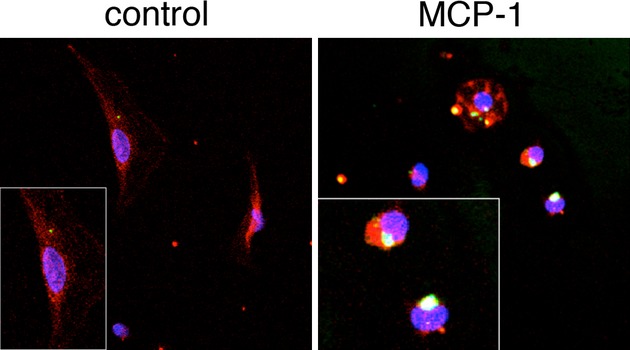
MCP‐1 causes the formation of intracellular MMP‐9 clusters in PF. Representative confocal micrographs are shown. PF were plated on glass coverslips 1 day after isolation and treated overnight with buffer alone or MCP‐1 (10 ng/mL). MMP‐9 immunostaining is pseudocolored green, rhodamine phalloidin staining (F‐actin) is pseudolored red, and TOPRO‐3 staining (nuclei) is pseudocolored blue. Scant MMP‐9 can be detected in untreated cells; however, increased numbers of large clusters of MMP‐9 fluorescence can be seen in PF treated with MCP‐1. Insets are digitally enhanced at 2X.

We investigated the effect of MCP‐1 on MMP‐9 localization within PF via confocal immunofluorescence (Fig. 3). No MMP‐9 protein could be detected in cells treated with buffer alone. In contrast, PF treated with MCP‐1 exhibited large clusters of MMP‐9 localized to the perinuclear cytoplasm, supporting the concept that MCP‐1 may block MMP‐9 protein release.

### Trafficking of exogenously expressed MMP‐9‐CFP is regulated by MCP‐1

**Figure 4.**
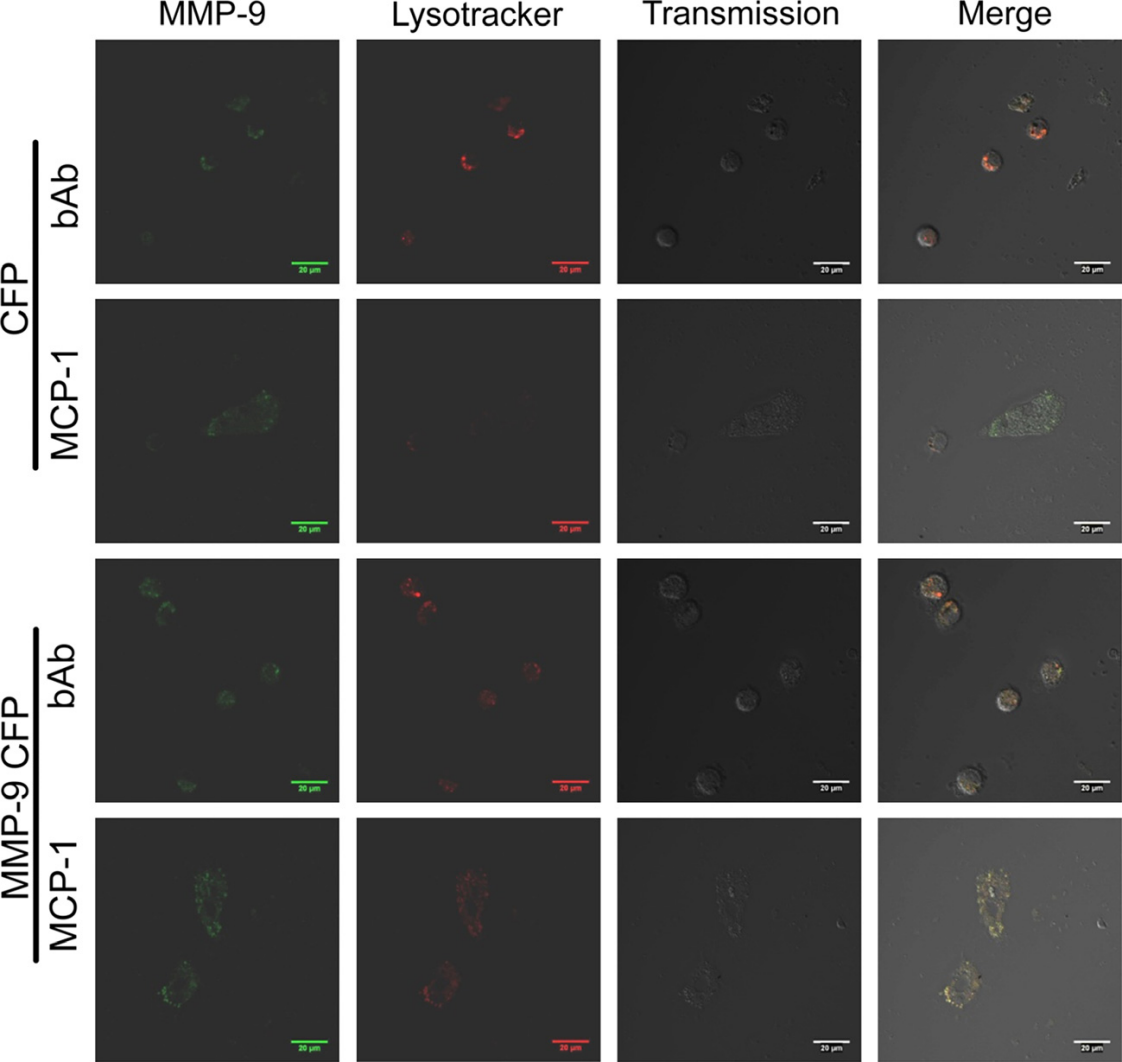
MCP‐1 causes MMP‐9‐CFP to be trafficked into lysosomes in live PF. PF were transfected with CFP vector alone (as a transfection control) or MMP‐9‐CFP. CFP fluorescence is pseudocolored green. Cells were incubated with Lysotracker Red (pseudocolored red) to visualize lysosomal membrane structures. No colocalization of CFP and Lysotracker was noted in cells treated with either MCP‐1 or MCP‐1 + blocking antibody (bAb). No colocalization of MMP‐9‐CFP with Lysotracker Red was seen in cells treated with MCP‐1 + bAb; however, strong colocalization MMP‐9‐CFP with Lysotracker Red was seen after MCP‐1 (10 ng/mL) treatment.

The effect of MCP‐1 in the presence or absence of MCP‐1 blocking antibody (bAb) (Kruglov et al. 2006) was assessed in live PF transfected with MMP‐9‐CFP and loaded with Lysotracker Red. Control cells were transfected with CFP alone. As seen in Figure 4, PF treated with MCP‐1 + bAb demonstrate MMP‐9‐CFP fluorescence in the perinuclear cytoplasm, but MMP‐9‐CFP fluorescence does not colocalize with Lysotracker Red fluorescence. In contrast, PF treated with MCP‐1 demonstrate MMP‐9‐CFP fluorescence in clusters within the cytoplasm that colocalize with Lysotracker Red. Taken together, this finding suggested that MCP‐1 induces trafficking of MMP‐9‐CFP to lysosomes.

### MCP‐1 induces trafficking of MMP‐9 to lysosomes

**Figure 5.**
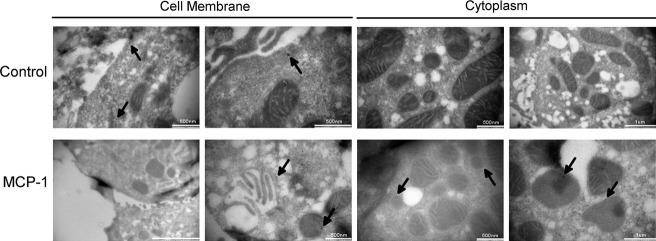
Effect of MCP‐1 on MMP‐9 localization as shown by immuno‐EM. PF were untreated or treated with MCP‐1 (10 ng/ml) overnight then fixed for EM processing. MMP‐9 was immunodetected with gold beads in PF fixed for EM imaging. In control cells, MMP‐9 is noted in a subplasmalemmar distribution and is absent from the cytoplasm. In contrast, cells treated with MCP‐1 (10 ng/mL) show minimal MMP‐9 expression near the plasma membrane but a large degree of MMP‐9 expression in round structures surrounded by single membranes, which are typical of lyososomes (Novikoff et al. 1971).

**Figure 6.**
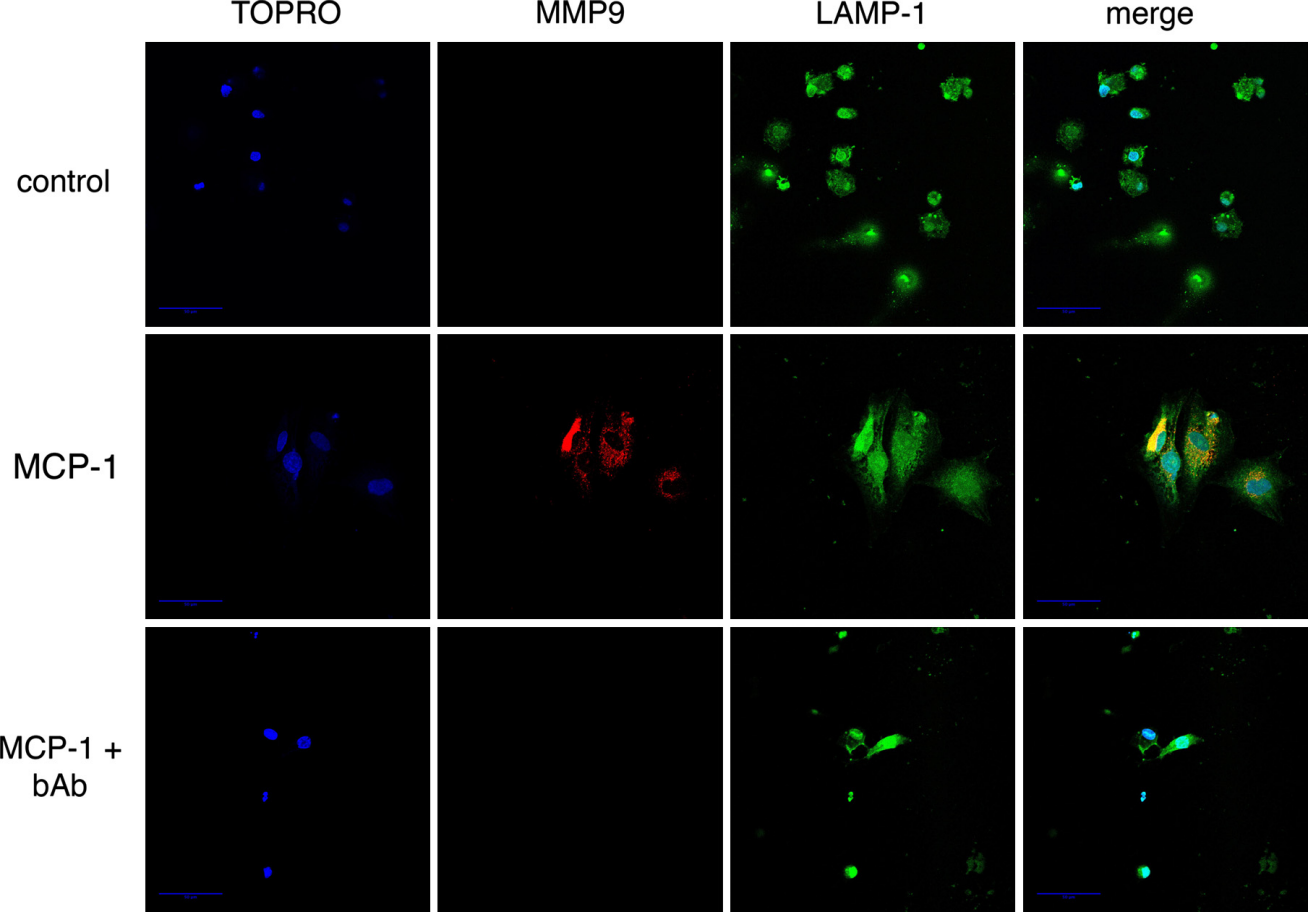
Effect of MCP‐1 on MMP‐9/LAMP‐1 colocalization in PF. PF were untreated or treated with MCP‐1 (10 ng/mL) ± blocking antibody (bAb) overnight then fixed. MMP‐9 and LAMP‐1 were detected by immunofluorescence, and nuclei were labeled with TOPRO. MMP‐9 fluorescence is pseudocolored red, LAMP‐1 fluorescence is pseudocolored green, and TOPRO fluorescence is pseudocolored green. In control cells or cells treated with MCP‐1 + bAb, no MMP‐9 intracellular fluorescence is noted. LAMP‐1 fluorescence is seen throughout the cell, with a concentration in the perinuclear cytoplasm. In contrast, PF treated with MCP‐1 demonstrate a large amount of MMP‐9 in a vesicular distribution, which overlies the LAMP‐1 fluorescence.

On the basis of the above observations, we attempted to determine directly whether MCP‐1 induced trafficking of endogenously expressed MMP‐9 to lysosomes. We did this using two separate approaches. First, we observed the distribution of MMP‐9 at the subcellular level in the presence or absence of MCP‐1 using immuno‐EM (Fig. 5). In control cells, MMP‐9 can be seen in vesicles near the plasma membrane; however, in PF treated with MCP‐1, MMP‐9 is seen in structures with typical features of lysosomes. Second, we determined whether MCP‐1 induced trafficking of MMP‐9 vesicles to regions expressing the lysosomal glyocoprotein LAMP‐1 (Eskelinen 2006) (Fig. 6). As noted previously in Figure 3, control cells or cells treated with MCP‐1 + bAb did not show MMP‐9 intracellular fluorescence. However, PF treated with MCP‐1 showed clusters of MMP‐9 in the perinuclear cytoplasm that colocalized with LAMP‐1. Taken together, these studies provided evidence that MCP‐1 shifts trafficking of MMP‐9 to lysosomes.

## Discussion

This study provides several findings worthy of further discussion. A summary of the new data gained is as follows: (1) MCP‐1 has physiologically relevant effects on PF, (2) MMP‐9 is downregulated in myofibroblastic PF, (3) the downregulation of MMP‐9 is posttranslational, (4) the mechanism for MMP‐9 regulation involves a shift of vesicular trafficking into a lysosome‐like compartment. Each of these concepts merits deeper consideration and will hopefully be a stimulus for study from the broader liver research community.

The concept that MCP‐1 has physiologically relevant effects on PF is not new. Several years ago, our group demonstrated that MCP‐1 upregulated proliferation, increased ASMA and procollagen‐1, and downregulated E‐NTPD2 in primary rat PF (Kruglov et al. 2006). Perhaps more importantly, that study suggested that the source of MCP‐1 in biliary cirrhosis is cholangiocytes. There is a growing body of literature demonstrating that cholangiocytes release a plethora of proinflammatory mediators in liver fibrosis, especially in the setting of biliary fibrosis/cirrhosis (Syal et al. 2012). Specific evidence that cholangiocytes produce MCP‐1 is now available (Jafri et al. 2009). Moreover, MCP‐1 appears to be particularly important in the pathogenesis of several human forms of biliary cirrhosis, including biliary atresia (Kobayashi et al. 2006) and primary biliary cirrhosis (Tsuneyama et al. 2001; Sasaki et al. 2014) and may even mediate the contributions of cholangiocytes in the nonbiliary cirrhosis occurring as a result of nonalcoholic fatty liver disease (Chiba et al. 2011). Thus, studies asking important questions about the target cells and effects of cholangiocyte‐secreted MCP‐1 are physiologically relevant.

The findings that PF downregulate MMP‐9 in a fashion that, while not unique, may be quite rare, is similarly noteworthy. First, MMP‐9 is a biologically relevant molecule in the pathogenesis of tissue/organ fibrosis (Yabluchanskiy et al. 2013). Although MMP‐9 is primarily regarded as a gelatinase (Vandooren et al. 2013), it also functions as a collagenase (in fact, it was first identified as such) (Xia et al. 1996), thus providing a mechanism for modulation of injury or scar resolution. Thus, fibrogenic cells may be predicted to downregulate MMP‐9 when producing matrix; however, this has not been examined in detail in liver myofibroblasts. It is interesting that the downregulation of MMP‐9 is entirely posttranslational, as so many genes relevant to liver fibrosis pathogenesis are regulated translationally. For example, we have examined changes in the ectonucleotidase NTPDase2 and the AMPase CD39, both of which are regulated completely at the transcriptional level via promoter elements relevant to fibrosis pathogenesis (Dranoff et al. 2002, 2004; Fausther et al. 2012). It is intriguing that MMP‐9 regulation, at least in PF, is “different”.

The mechanism of regulation of plasma membrane protein content via shunting to lysosomes is well‐reported (Bradbury 1999); however, evidence of shunting of secreted proteins to lysosomes is rare (Evans et al. 2011; Manickam et al. 2011). We have hesitated to call the structures to which MMP‐9 is shunted lysosomes; however, they have single membrane structures (Fig. 5) and express lysosomal proteins and lipids (Figs 4, 6). Thus, it is highly likely that these structures are indeed lysosomes. Moreover, the effect observed holds true both for endogenous MMP‐9 and overexpressed MMP‐9‐CFP. If MMP‐9 shunting to lysosomes is verified to be the case in future studies, it will be necessary to examine modifications of the MMP‐9 protein as it traverses the secretory apparatus in quiescent and myofibroblastic PF. One may predict myofibroblastic PF and other liver myofibroblasts may use similar protein modifications to change their secretory load, ultimately resulting in profibrogenic cells.

In summary, PF regulate MMP‐9 in a relatively novel fashion in the setting of myofibroblastic differentiation. Future studies will be necessary to verify these findings, test whether they are applicable to other scar‐producing cells in and outside of the liver, and to discover the posttranslational changes that occur to allow shunting of secretory vesicles.

## Conflict of Interest

None declared.
